# Genomic surveillance of invasive *Streptococcus pneumoniae* isolates in the period pre-PCV10 and post-PCV10 introduction in Brazil

**DOI:** 10.1099/mgen.0.000635

**Published:** 2021-10-05

**Authors:** Samanta C. G. Almeida, Stephanie W. Lo, Paulina A. Hawkins, Rebecca A. Gladstone, Ana Paula Cassiolato, Keith P. Klugman, Robert F. Breiman, Stephen D. Bentley, Lesley McGee, Maria-Cristina de C. Brandileone

**Affiliations:** ^1^​ National Reference Laboratory for Meningitis and Pneumococcal Infections, Institute Adolfo Lutz, São Paulo, Brazil; ^2^​ Parasites and Microbes Programme, Wellcome Sanger Institute, Hinxton, UK; ^3^​ Respiratory Diseases Branch, Centers for Disease Control and Prevention, Atlanta, USA; ^4^​ Emeritus Professor of Global Health, Emory University, Atlanta, GA, USA; ^5^​ Emory Global Health Institute, Emory University, Atlanta, USA

**Keywords:** Brazil, genomic surveillance, global pneumococcal sequence cluster, Multi-locus sequencing typing, PCV10, *Streptococcus pneumoniae*

## Abstract

In 2010, Brazil introduced the 10-valent pneumococcal conjugate vaccine (PCV10) into the national children’s immunization programme. This study describes the genetic characteristics of invasive *Streptococcus pneumoniae* isolates before and after PCV10 introduction. A subset of 466 [pre-PCV10 (2008–2009): *n*=232, post-PCV10 (2012–2013): *n*=234;<5 years old: *n*=310, ≥5 years old: *n*=156] pneumococcal isolates, collected through national laboratory surveillance, were whole-genome sequenced (WGS) to determine serotype, pilus locus, antimicrobial resistance and genetic lineages. Following PCV10 introduction, in the <5 years age group, non-vaccine serotypes (NVT) serotype 3 and serotype 19A were the most frequent, and serotypes 12F, 8 and 9 N in the ≥5 years old group. The study identified 65 Global Pneumococcal Sequence Clusters (GPSCs): 49 (88 %) were GPSCs previously described and 16 (12 %) were Brazilian clusters. In total, 36 GPSCs (55 %) were NVT lineages, 18 (28 %) vaccine serotypes (VT) and 11 (17 %) were both VT and NVT lineages. In both sampling periods, the most frequent lineage was GPSC6 (CC156, serotypes 14/9V). In the <5 years old group, a decrease in penicillin (*P*=0.0123) and cotrimoxazole (*P*<0.0001) resistance and an increase in tetracycline (*P*=0.019) were observed. Penicillin nonsusceptibility was predicted in 40 % of the isolates; 127 PBP combinations were identified (51 predicted MIC≥0.125 mg l^−1^); cotrimoxazole (*fol*A and/or *fol*P alterations), macrolide (*mef* and/or *ermB*) and tetracycline (*tet*M, *tet*O or *tet*S*/*M) resistance were predicted in 63, 13 and 21.6 % of pneumococci studied, respectively. The main lineages associated with multidrug resistance in the post-PCV10 period were composed of NVT, GPSC1 (CC320, serotype 19A), and GPSC47 (ST386, serotype 6C). The study provides a baseline for future comparisons and identified important NVT lineages in the post-PCV10 period in Brazil.

## Data Summary

Genome sequences are deposited in the European Nucleotide Archive (ENA), the accession number and the sample data is available in the supplementary material (Data_Summary_GPS_Brazil.xlsx). The authors confirm all supporting data, code and protocols have been provided within the article or through supplementary data files.

**Impact Statement**

This study, based on WGS analysis, makes several noteworthy contributions to understanding the genetic structure of the *S. pneumoniae* population in Brazil. We analysed genomic data from invasive pneumococcal isolates collected in Brazil between 2008 and 2013 to provide a detailed description of the population structure during that sampling period. We identified globally spreading lineages that also included non-vaccine serotype components, indicating that they potentially might contribute to vaccine evasion. The data generated by this study can be used as a baseline to determine vaccine impact during the following years.

## Introduction

*Streptococcus pneumoniae* is the main cause of otitis media and community-acquired pneumonia as well as invasive pneumococcal disease (IPD), including meningitis, sepsis and bacteremia [1]. Antimicrobials and vaccines are tools currently available to treat and prevent pneumococcal diseases, respectively.

The indiscriminate use of antimicrobial agents in community settings results in the selection of resistant pneumococcal strains and impacts IPD by resulting in antibiotic treatment failure [2]. In addition, pressure due to vaccination can change resistance patterns temporally and geographically [1].

Pneumococcal conjugate vaccines (PCV, 7-valent PCV, 10-valent PCV, and 13-valent PCV) are highly effective in preventing IPD caused by serotypes present in its composition [3, 4]. Brazil was the first country to introduce the 10-valent pneumococcal conjugate vaccine (PCV10, target serotypes 1, 4, 5, 6B, 7F, 9V, 14, 18C, 19F and 23F) into their national childhood immunization programme in March 2010 [5]. The vaccine schedule was three primary doses at ages 2, 4 and 6 months and a booster dose for children aged 12–15 months. During the first year of PCV10 introduction, a catch-up campaign with two primary doses for children at 7 to 11 months of age plus a booster dose at 12–15 months, and a single dose for children aged 12 to <24 months was adopted [5]. In 2016, the primary schedule was changed to two primary doses at ages 4 and 6 months and a booster dose at 12 months [6].

It is well documented that PCV introduction was followed by changes in *S. pneumoniae* epidemiology due to (i) vaccine pressure leading to evidence of serotype replacement by the capsular switch, (ii) through the expansion of common strains and (iii) by increases in newly emerging non-vaccine type strains [3, 4]; as well as reductions in the transmission of vaccine types (VT) resulting in the indirect effect of herd immunity in the unvaccinated population. Therefore, surveillance is essential to detect potential temporal changes in the epidemiological and genetic characteristics of pneumococcal isolates [1, 7, 8]. Genomic methods have proved to be excellent tools for understanding the biology and epidemiology of important bacterial pathogens, including *S. pneumoniae *. Multi-locus sequence typing (MLST) is a molecular method historically important, and still widely used in pneumococcal epidemiology that consists of sequencing seven housekeeping genes as a sample of genomic variation and is used to define the sequence type (ST) and clonal complexes (CCs) [1]. This present study aimed to describe the genetic characteristics of invasive pneumococcal disease (IPD) isolates sampled from ongoing routine laboratory surveillance in Brazil during the pre- (2008–2009) and post- (2012–2013) PCV10 introduction periods. The whole-genome sequence data was used for *in silico* analysis of the serotype, antimicrobial resistance predictions, to identify possible changes in genetic lineages, and circulation of multi-drug-resistant clones following PCV10 introduction. The data generated will provide a baseline for continued vaccine impact monitoring and support future vaccination strategies for pneumococcal disease control.

## Methods

### Bacterial strain collection

The study collection consisted of a random subset of 466 IPD isolates recovered through a national laboratory surveillance network led by Institute Adolfo Lutz (IAL), the Brazilian National Reference Laboratory for Meningitis and Pneumococcal Infections. This study included pneumococcal isolates from pre- (*n*=232, 2008–2009) and post- (*n*=234, 2012–2013) PCV10 introduction periods collected in 20 of 26 Brazilian States (Table S1, available in the online version of this article) and corresponding to 14 % (*n*=466/3342) of the total *S. pneumoniae* isolates received by IAL in the period analysed. Isolates from the years 2010 and 2011 corresponding to the first years of PCV10 introduction were excluded from the study. Table S2 shows the pneumococcal study collection isolates stratified by age groups, clinical diagnosis and vaccine periods. Brandileone and collaborators [9] included in their publication the detailed phenotypical analyses of the pneumococcal serotypes that caused IPD before and after the introduction of PCV10 using data from the laboratory surveillance system in Brazil from a larger period (2005 to 2015) of time. From this dataset, data of 3342 isolates corresponding to the periods from 2008 to 2009 and 2012 to 2013 were used in our study as a basis for selection and comparison of serotype distribution of the 466 isolates whole-genome sequencing (WGS) subset (Figs S1 and S2). We presented serotype data for the entire collection of 3342 isolates and restrict other analyses to the 466 randomly sampled WGS.

The IAL receives strains previously identified as *S. pneumoniae* by the laboratory of origin and confirms this identification using classical methodologies described by WHO [10]. For routine surveillance, serotyping was performed by Quellung reaction and antimicrobial susceptibility profiles were determined by the disc diffusion and/or broth microdilution to determine minimal inhibitory concentrations (MIC) according to Clinical Laboratory Standards Institute (CLSI) breakpoints [11–13].

### Genome sequencing and analyses

The 466 IPD isolates were WGS on the Illumina HiSeq platform to produce paired-end reads of 150 base pairs in length and raw data were deposited in the European Nucleotide Archive (ENA) (Supplementary Material: Data_Summary_GPS_Brazil.xlsx). WGS data were processed as previously described [14]. We derived the virulence factors (serotype [15] and pilus locus [16]) and multi-locus sequencing types (STs) [17].

The genetic structure was defined by assigning the clonal complexes (CCs) from the STs previously described by the Global Pneumococcal Sequencing Project (GPS) [14] and also by assigning Global Pneumococcal Sequence Cluster (GPSC) on each isolate using a PopPUNK [18], along with a reference list of pneumococcal isolates (*n*=13 454) in the GPS database (https://www.pneumogen.net/gps/assigningGPSCs.html). The STs and GPSCs described in this study were deposited in the PubMLST (https://pubmlst.org/organisms/streptococcus-pneumoniae) and GPS databases (https://www.pneumogen.net/gps/assigningGPSCs.html), respectively. Phylogenetic analysis was performed on all Brazilian isolates in this study by constructing a maximum-likelihood tree using FastTree [19]. In brief, the tree was built upon a SNP alignment after mapping reads to the reference genome of *S. pneumoniae* ATCC 700669 (NCBI accession number FM211187) using Burroughs Wheeler Aligner (BWA).

Capsular or serotype switching was identified in isolates with identical ST but different serotypes in this study. For each ST, we examined the genetic relatedness of isolates in lineage-specific phylogenies and place the Brazilian lineage of interest in a global context by including other GPS published isolates belonging to the same GPSC [20]. The lineage-specific tree was constructed using GUBBINS [21]. In brief, GUBBINS detects recombination regions and removes them when constructing the phylogeny. The recombination-free phylogeny created by GUBBINS was used as input for BactDating [22], an R package used to create a time-measured phylogeny performing Bayesian dating inference of the nodes on the bacterial phylogenetic tree; typically involves simultaneous Bayesian estimation of the molecular clock rate and coalescent rate as previously described [20]. The time-measured tree was used to estimate the period when capsular switching occurred.

Resistance profiles for six antibiotics, including penicillin [defined as penicillin binding protein transpeptidase amino acid sequence types (PBP types) based on *pbp*1A, *pbp*2B, *pbp*2X changes] [23, 24], chloramphenicol (*cat*), cotrimoxazole (*fol*A and *fol*P), erythromycin (*erm*B and *me*fA), tetracycline (*tet*M, *tet*O and *tet*S/M), and vancomycin (*vanA*, *van*B, *van*C, *van*D, *van*E and *vanG*) were predicted from genomic data, as previously described [16, 25]. Multidrug resistance (MDR) was defined as isolates with predicted intermediate resistance or resistance to three or more classes of antibiotics [26]. The serotype and antimicrobial resistance predictions for the 466 sampled isolates were compared to the phenotypic results generated from routine surveillance.

### Statistical analyses

The pneumococcal isolates were defined as vaccine serotype (VT) when isolates belonged to predicted serotypes included in PCV10 (1, 4, 5, 6B, 7F, 9V, 14, 18C, 19F and 23F), and as non-vaccine serotypes (NVT) for the predicted serotypes non-PCV10, including the additional PCV13 serotypes 3, 6A and 19A. We defined the status of a lineage (GPSC) as VT (100 % PCV10 serotypes), NVT (100 % non-PCV10 serotypes) and GPSC with both VT and NVT isolates, based on its serotype composition detected in the whole study period. The prevalence of *in silico* serotypes was stratified by age groups (<5 years old and ≥5 years old).

As the population denominators are unavailable, we evaluated significant changes of VT/NVT in each GPSC lineage in proportion to all VT/NVT, respectively, using Fisher’s exact test. This calculation was performed to avoid the overestimation of the NVT increase. Overall, and by GPSC, the prevalence of antibiotic resistance between vaccine periods was also detected using Fisher’s exact test. Two-sided *P*-values of <0.05 were considered statistically significant. The number of samples was calculated to achieve 80 % of statistical power with a significant level of *P*-values. Before using the Fisher’s exact tests to compare variables (e.g. VT or penicillin resistance) before and after the PCV10 period, we calculated the number of samples that we need to achieve an 80 % statistical power with a significant level of *P*-value<0.05 using the R package pwr, which contains functions for basic power calculation [27]. When the variables (VT and/or antibiotic resistance) would not have sufficient statistical power to be tested, Fisher’s exact test was not performed. Multiple testing was adjusted using the Benjamin-Hochberg false discovery rate of 5 %, the statistical analysis was carried out in R version 3.5.2, and R scripts used for analyses were deposited at GitHub (https://github.com/StephanieWLo/Genomic-Surveillance).

## Results

### Serotype distribution

No discrepancies were observed between the 466 predicted serotypes and the Quellung results, and the frequency of the predicted serotypes in our study subset reflected the frequency of the serotypes identified in the larger 3342 isolates' collection. Figs S1 and S2 show serotype distribution by vaccine period (pre-PCV10 and post-PCV10), for age groups <5 years and ≥5 years, and for the larger and subset collections included in this WGS study.

As expected, a higher number of VT isolates was observed in the pre-PCV10 period mainly in the <5 years while NVT (including the additional PCV13, 3, 6A and 19A) were more frequent in the post-PCV10 period (Fig. S1). Before vaccination, serotype 14 was highly common in both age groups and after vaccine introduction, serotype 3 was most frequent in children aged <5 years, followed by serotype 19A, 6A, 12F and 6C (Fig. S1), and in ≥5 years serotype 12F, 3, as well the serotypes 8 and 9 N (Fig. S2).

### Pneumococcal lineages

The predictions of serotype, ST and GPSC by age group and vaccine period of the 466 *S *. *pneumoniae* invasive study isolates are presented in Table S3.

A total of 159 STs were identified among 466 isolates sequenced, belonging to 54 CCs and 42 singletons. The phylogeny supported the good correlation of the CCs with the WGS-based GPSCs and, the latter typing scheme, revealed the genetic relatedness among the CCs 66, 81, 2216, 9747 plus three singletons (ST4913, 12483, 13878) in the GPSC16, one of the major GPSCs identified in the study (Fig. S3).

Overall, 65 GPSCs were identified and the most prevalent GPSCs were GPSC6 (CC156, serotype 14/9V), GPSC16 (CC66/81/2216/9747 and ST4913/12483/13878, serotypes 7C/9 N/14/15A/19F/23F/24/40), GPSC12 (CC180, serotype 3), GPSC5 (CC172, serotypes 6C/15B/15C/23A/23F) and GPSC11 (CC193, serotypes 11A/15A/15B/15C/18B/18C) (Fig. S3). Of the GPSCs identified, 36 GPSCs (55%) belonged to NVT lineages, 18 GPSCs (28 %) to VT lineages, and 11 (17 %) included both VT and NVT lineages (GPSC1, 5, 10, 11, 13, 16, 18, 23, 47, 61 and 231). Among the 466 pneumococcal isolates, 410 (88 %) were assigned to 49 GPSCs that have previously been found in the GPS reference database (last updated in April 2019, *n*=20 187, www.pneumogen.net\\gps\\assigningGPSCs.html) the remain 16 GPSCs (204, 231, 249, 289, 311, 341, 392-394, 571, 573-575, 577, 702, 811) included STs assigned in the international PubMLST database as mainly associated with Brazil (http://pubmlst.org, accessed: 26/05/2021) (Table S3). All eight global-spreading lineages recognized in the previous GPS study [14] were found in the current bacterial collection with an overall prevalence of 44.9 %: 17.8 % GPSC6, 9.0 % GPSC16, 5.6 % GPSC12, 3.9 % GPSC1, 3.0 % GPSC32, 2.6 % GPSC18, 1.5 % GPSC7, and 1.5 % GPSC23 (Table S3).

**Fig. 1.**
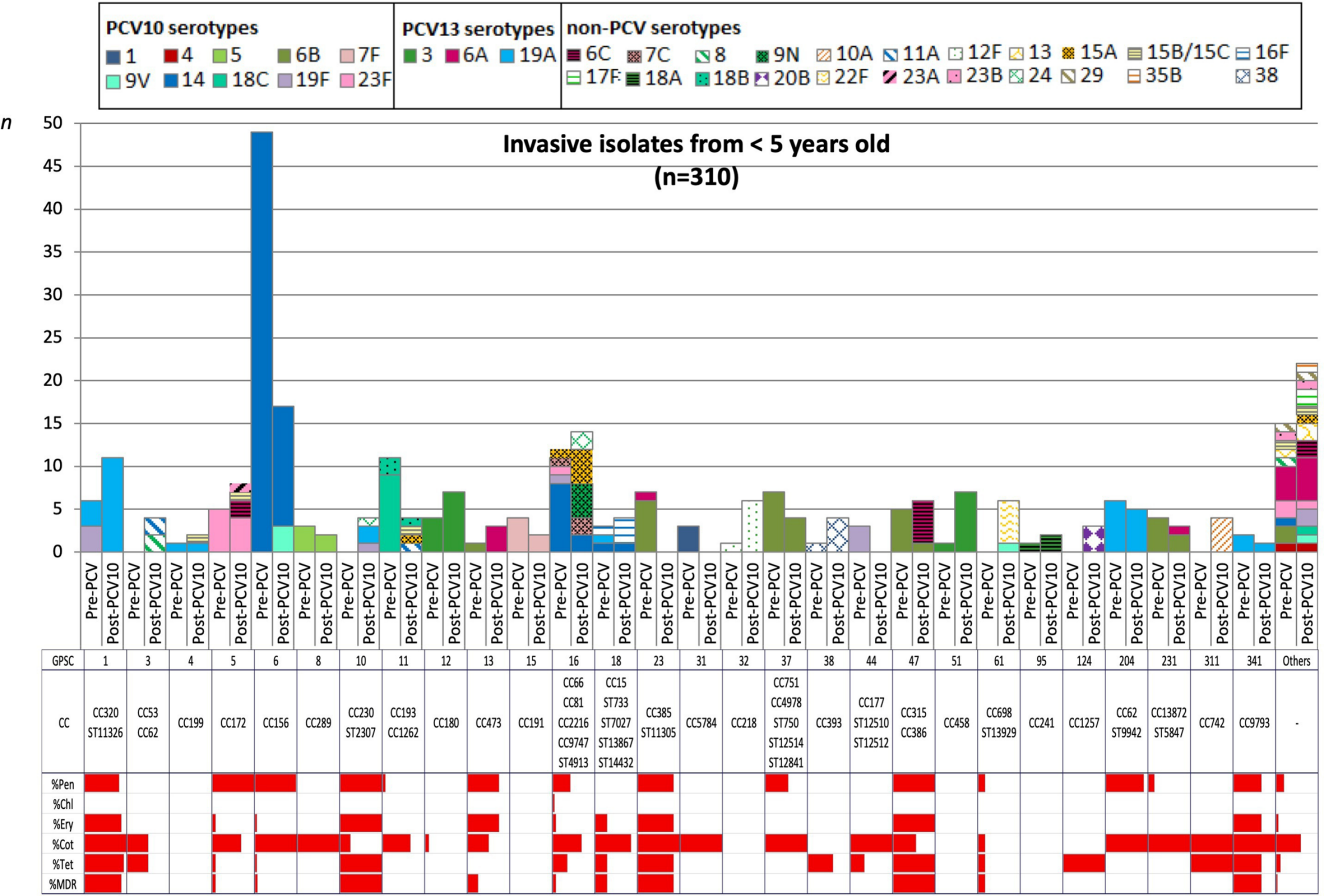
Dynamics of Global Pneumococcal Sequence Clusters (GPSCs) among invasive isolates from children aged <5 years old over vaccine periods in Brazil. The number of invasive pneumococcal isolates, coloured by serotypes, is plotted by GPSC with stratification into two vaccine periods (pre-PCV10 and post-PCV10) and MLST CC. Solid fill represented the VT and NVT additional PCV13 serotypes while hatched patterns represented the NVT non-PCV serotypes. The antibiotic resistance pattern to penicillin, chloramphenicol, erythromycin, cotrimoxazole, tetracycline and MDR are present for each GPSC and in the entire period studied.

**Fig. 2.**
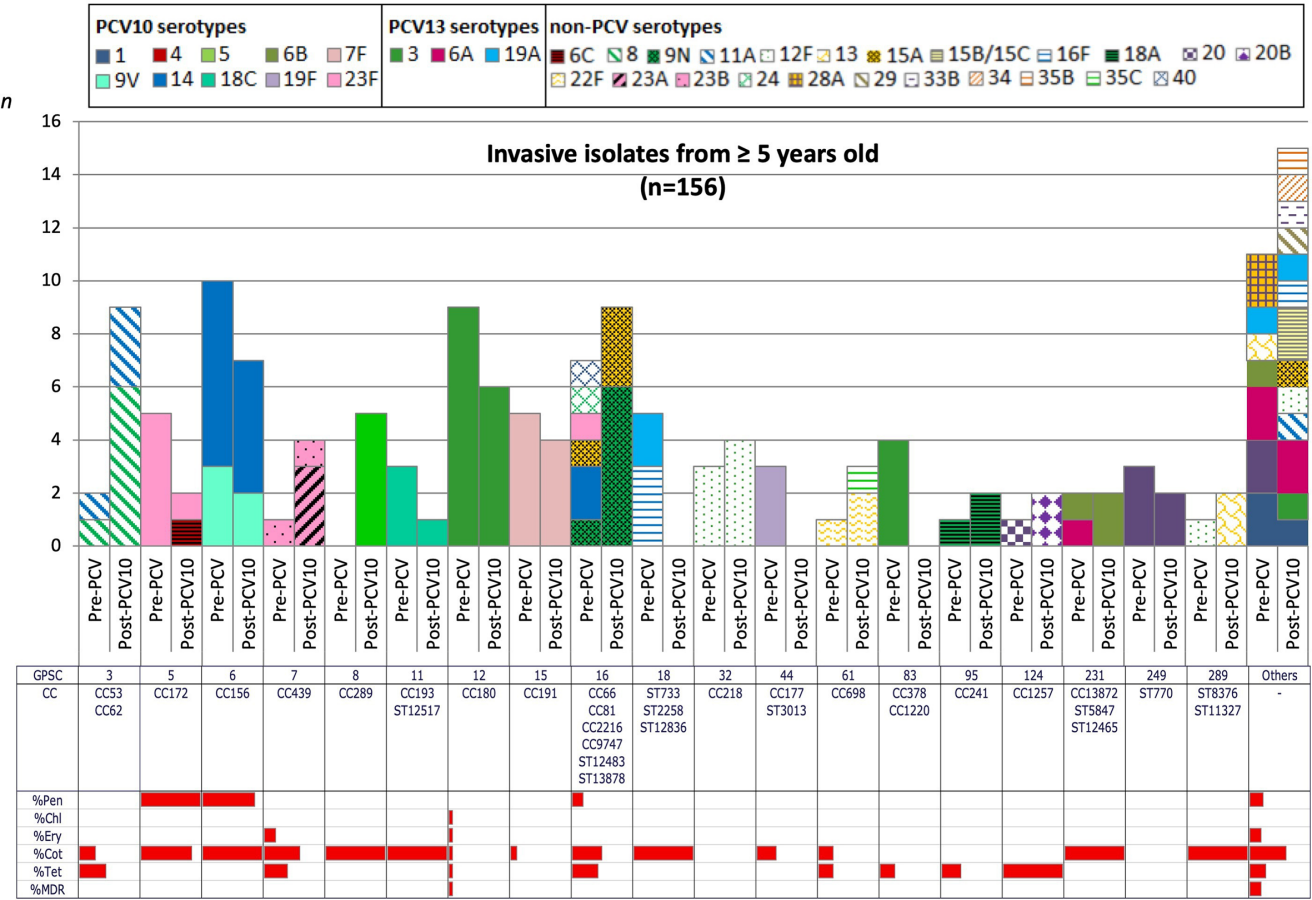
Dynamics of Global Pneumococcal Sequence Clusters (GPSCs) among invasive isolates from children aged ≥5 years old over vaccine periods in Brazil. The number of invasive pneumococcal isolates, coloured by serotypes, is plotted by GPSC with stratification into two vaccine periods (pre-PC10 and post-PCV10) and MLST CC. Solid fill represented the VT and NVT additional PCV13 serotypes while hatched patterns represented the NVT non-PCV serotypes. The antibiotic resistance pattern to penicillin, chloramphenicol, erythromycin, cotrimoxazole, tetracycline and MDR are present for each GPSC and in the entire period studied.

In comparison with the pre-PCV10 period, we detected any significant changes in the frequency of VT and NVT within GPSC, but we do not have sufficient statistical power to detect changes in each serotype. However, isolates associated with VT GPSCs decreased from 48–20 % (*P*<0.0001) and 32–24 % (*P*=0.2871) in the age groups <5 and ≥5 years old respectively, while isolates belonging to NVT GPSCs increased from 17–39 % (*P*<0.0001) and 34–52 % (*P*=0.0246). The five most frequent GPSCs by age groups are listed in Tables 1 and 2. GPSC6 and GPSC16 were among the top lineages in both age groups, pre-PCV10 and post-PCV10 periods. Though GPSC6, composed of CC156 and VT 9V and 14, remained a predominant lineage during the whole period of study and showed a decreasing trend among the <5 years old group (Fig. S4). In contrast, GPSC16 lineage persisted with the NVT components 9 N and 15A frequently observed in the post-PCV10 period. In the post-PCV10 NVT lineages were mainly associated with children aged <5 years; GPSC1 (CC320, serotype 19A), GPSC12 (CC180, serotype 3) and GPSC51 (CC458, serotype 3) (Fig. 1, Table 1). In the ≥5 years old group, GPSC3 expressing serotypes 8 (CC53) and 11A (CC62) became the predominant lineage 2–3 years after PCV10 introduction, though it was not in the top five lineages before vaccine roll-out (Fig. 2, Table 2).

**Table 1.** The five most frequent lineages associated with serotypes of IPD isolates in the age group **<**5 years old (*N*=310) in the pre-PCV10 (2008–2009) and the post-PCV10 periods (2012–2013), Brazil

Rank	Pre-PCV10 period (*n*=155)			Post-PCV10 period (*n*=155)		
	GPSC (CC)	*N (%)*	Associated serotypes (*n*)^*a*^	GPSC (CC)	*N* (%)	Associated serotypes (*n*)^*a*^
First	GPSC6 (CC156)	49 (32 %)	14 (49)	GPSC6 (CC156)	17 (11 %)	14 (14), 9V (3)
Second	GPSC16 (CC66, CC81)	12 (8 %)	14 (8), 7 C (1), 15A (1), 19F (1), 23F (1)	GPSC16 (CC66, ST4913, CC2216, CC9747)	14 (9 %)	9 N (4), 15A (4), 7 C (2), 14 (2), 24 (2)
Third	GPSC11 (CC193)	11 (7 %)	18C (9), 18B (2)	GPSC1 (CC320)	11 (7 %)	19A (11)
Fourth	GPSC23 (CC385, ST11305)	7 (5 %)	6B (6), 6A (1)	GPSC5 (CC172)	8 (5 %)	23F (4), 6 C (2), 15B/ 15 C (1), 23A (1)
	GPSC37 (CC751, CC4978, ST750, ST12514, ST12841)	7 (5 %)	6B (7)			
Fifth	GPSC1 (CC320, ST11326)	6 (4 %)	19A (3), 19F (3)	GPSC12 (CC180)	7 (4,5 %)	3 (7)
	GPSC204 (CC62, ST9942)	6 (4 %)	19A (6)	GPSC51 (CC458)	7 (4,5 %)	3 (3)

*a,* Black, VT or PCV10 serotypes; blue, NVT additional PCV13 serotypes; and red, NVT non-PCV serotypes.

**Table 2.** The five most frequent lineages associated with serotypes of IPD isolates in the age group **≥**5 years old (*N*=156) in the pre-PCV10 (2008–2009) and the post-PCV10 periods (2012–2013), Brazil

Rank	Pre-PCV10 period (*n*=77)			Post-PCV10 period (*n*=79)		
	GPSC (CC)	*N* (%)	Associated serotypes (*n*)^*a*^	GPSC (CC)	*N* (%)	Associated serotypes (*n*)^*a*^
First	GPSC6 (CC156)	10 (13 %)	14 (7), 9V (3)	GPSC3 (CC53, CC62)	9 (11 %)	8 (6), 11A (3)
				GPSC16 (CC66, CC2216, ST13878)	9 (11 %)	9 N (6), 15A (3)
Second	GPSC12 (CC180)	9 (12 %)	3 (9)	GPSC6 (CC156)	7 (9 %)	14 (5), 9V (2)
Third	GPSC16 (CC66, CC2216, CC81, CC9747, ST12483)	7 (9 %)	14 (2), 9N (1), 15A (1), 23F (1), 24 (1), 40 (1)	GPSC12 (CC180)	6 (8 %)	3 (6)
Fourth	GPSC5 (CC172)	5 (6 %)	23F (5)	GPSC8 (CC289)	5 (6 %)	5 (5)
	GPSC15 (CC191)	5 (6 %)	7F (5)			
	GPSC18 (ST2258, ST12836, ST733)	5 (6 %)	16 F (3), 19A (2)			
Fifth	GPSC83 (CC378, CC1220)	4 (5 %)	3 (4)	GPSC7 (CC439)	4 (5 %)	23A (3), 23B (1)
				GPSC15 (CC191)	4 (5 %)	7F (4)
				GPSC32 (CC218)	4 (5 %)	12 F (4)

*a*, Black, VT or PCV10 serotypes; blue, NVT additional PCV13 serotypes; and red, NVT non-PCV serotypes.

### Capsular switch variants

Among the STs identified, nine occurred in more than one serotype and are suggestive of capsular switching events: STs 66, 156, 193, 199, 338 and 386; four are NVT switches and were observed in the post-PCV10 period (ST66 serotype 9 N, ST 199 serotypes 19A/15B/15C, ST338 serotype 15B/15C, and ST386 serotype 6C).

**Fig. 3.**
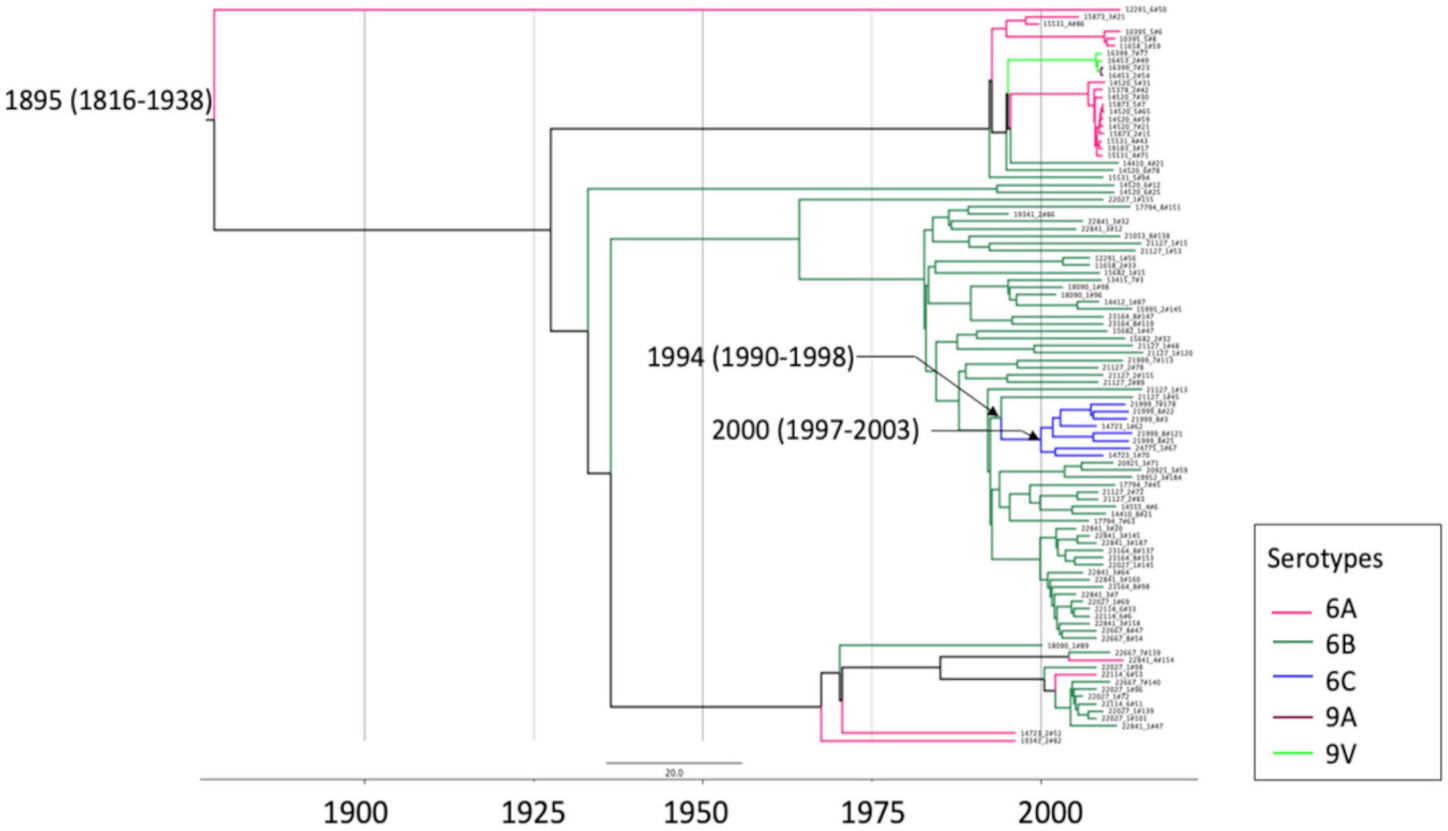
A timed-measured phylogeny of GPSC47 (ST386) isolates from Brazil and the other 15 countries from Gladstone *et al.* [14]. The phylogeny is built using BactDating [22] with 100 000 000 generations on a recombination-free SNP alignment generated by GUBBINs [21]. The most recent common ancestor (tMRCA) of the serotype 6C clade (all isolates are ST386) is estimated to emerge in around 2000 (95 % confidence interval: 1997–2003), and the capsular switching occurred among 1990–2003. The phylogeny and metadata can be interactively visualized at https://microreactorg/project/tmxT66fLq56uZb5VfA1BYC.

The ST386 (GPSC47) was represented by serotypes 6B in the pre-PCV10 period and 6C in the post-PCV10 period (Fig. 1) and a time-measured phylogeny of GPSC47 using isolates from the GPS database and including the ST386 of this study, showed serotype 6C grouped separately from the ST386 serotype 6B isolates (Fig. 3). Using BactDating software we estimated a possible capsular switch may have occurred between serotypes 6B and 6C isolates around 1994 (95 % confidence interval: 1990–1997). This suggests the capsular switch event occurred in the pre-PCV era leading to the selection of the NVT lineage GPSC47 (ST386, serotype 6C) over the VT lineage GPSC47 (ST386, serotype 6B) following PCV introduction.

### Antimicrobial resistance

The antimicrobial susceptibility testing performed at IAL showed good correlation (susceptible, intermediate or resistant category) with the *in silico* prediction, presenting a 100 % of the agreement for erythromycin, chloramphenicol and vancomycin; 98.9 % (461/466) for tetracycline, 98.7 %(460/466) for penicillin and 93.6 %(436/466) for cotrimoxazole. Due to the high level of concordance between phenotype and genotype results, we used the WGS predicted antimicrobial resistance in the following analyses.

The antimicrobial non-susceptibility patterns between pre-and post-PCV10 periods showed a significant increase of tetracycline resistance (*P*=0.0019) and a decrease of penicillin (*P*=0.0123) and cotrimoxazole resistance (*P*<0.0001) among isolates from children aged <5 years after vaccination. No significant difference (*P*≥0.05) was observed in the frequency of predicted non-susceptibility to chloramphenicol, erythromycin and MDR isolates from <5 years, as well for all studied antibiotics in the isolates from ≥5 years (Table 3). All isolates were predicted as vancomycin susceptible.

**Table 3.** Proportions of IPD isolates with antibiotic non-susceptibility in pre-PCV10 and post-PCV10 periods (*N*=466), Brazil

Antibiotics^*a*^	Age <5 years old			Age ≥5 years old		
	Pre-PCV10 (*N*=155)	Post-PCV10 (*N*=155)	*P*-value^*b*^	Pre-PCV10 (*N*=77)	Post-PCV10 (*N*=79)	*P*-value^*b*^
Penicillin	88 (57 %)	66 (42 %)	**0.0123**	18 (23 %)	17 (22 %)	0.8488
Chloramphenicol	1 (0.6 %)	0	1.0000	1 (1 %)	1 (1 %)	1.0000
Cotrimoxazole	133 (86 %)	81 (52 %)	**<0.0001**	42 (55 %)	37 (47 %)	0.3426
Erythromycin	19 (12 %)	32 (21 %)	0.0653	3 (4 %)	5 (6 %)	0.7195
Tetracycline	24 (15 %)	48 (31 %)	**0.0019**	11 (14 %)	17 (22 %)	0.2982
MDR^*c*^	19 (12 %)	31 (20 %)	0.0887	3 (4 %)	4 (5 %)	1.0000

*a,* CLSI, breakpoints: penicillin, susceptible ≤0.06 mg l^−1^ and resistant ≥0.125 mg l^−1^; chloramphenicol, susceptible ≤4 mg l^−1^ and resistant ≥8 mg l^−1^; cotrimoxazole, susceptible ≤0.5/9.5 mg l^−1^, intermediate 1/19-2/38 mg l^−1^ and resistant ≥4/76 mg l^−1^; erythromycin, susceptible ≤0.25 mg l^−1^, intermediate 0.5 mg l^−1^ and resistant ≥1 mg l^−1^; and tetracycline, susceptible ≤1 mg l^−1^, intermediate 2 mg l^−1^, and resistant ≥4 mg l^−1^.

*b*, Fisher´s two-tailed test.

*c*, MDR, multidrug resistance; intermediate or resistant isolates to three or more classes of antibiotic.

WGS analysis identified 127 PBP types allele combinations, 51 of them predicted non-susceptibility to penicillin (MIC ≥0.125 mg l^−1^). Independent of age group, the PBP allele combinations 13-11-16 (*n*=12, MIC=4 mg l^−1^), 15-12-18 (*n*=11, MIC=2 mg l^−1^) and 45-12-63 (*n*=8, MIC=2 mg l^−1^) were predominant and associated with specific international antimicrobial-resistant lineages, GPSC1 (CC320, serotype 19A), GPSC6 (CC156, serotypes 9V) and GPSC6 (CC156, serotype 14), respectively. Despite the other PBP profiles that confer penicillin resistance in the post-PCV10 period, we highlight the third most frequent PBP profile in the <5 years old group the profile 2-53-77 (*n*=6, MIC=0.125 mg l^−1^) observed in the lineage GPSC47 (ST386, serotype 6C and CC315, serotype 6B) since it is associated with an important NVT and capsular switching described in our study (Table S4).

The frequency of the gene combination *erm*B and *mef* increased from 2 (1 %) to 11 (7 %) isolates in the pre-PCV10 vs post-PCV10 period in the <5 years old and was associated with GPSC1 (CC320, serotype 19A). Full resistance to cotrimoxazole (MIC ≥4 mg l^−1^) was characterized for the presence of alterations in genes *fol*A (all isolates have the I100L substitution) and *fol*P, which were detected in a large proportion of the isolates (*n*=210, 46 %). As previously shown [16], a mutation within *folA* or *folP* alone conferred intermediate cotrimoxazole resistance, while mutations within both *folA* (I100L) and *folP* (1–2 codon insertions) conferred full resistance. For tetracycline, the most frequent resistance gene detected was *tet*M (*n*=97, 21 %), mainly identified in the post-PCV10 period (*n*=62/234, 26 %) and associated with GPSC1 (CC320, serotype 19A) and GPSC16 (CC66, serotype 9 N). Additionally, we observed lower frequencies of *tet*O and the combination of the *tet*S and *tet*M genes also predicting tetracycline resistance. The *cat* gene conferring chloramphenicol resistance, substitutions in the *rpo*B gene (P15A, H21N, or K22N) that predict rifampicin resistance (MIC >2 mg l^−1^) and substitutions in the *par*C gene (S79C, S29F, or S79Y) predicting fluoroquinolone resistance were identified in only a few isolates (Table 4).

**Table 4.** Non-penicillin resistance gene determinants from IPD isolates by age groups in pre-PCV10 and post-PCV10 periods, Brazil

Antibiotic	Resistance genes	Age <5 years old (*N*=310)		Age≥5 years old (*N*=156)		Total (*N*=466)
		Pre-PCV10 (*n*=155)	Post-PCV10 (*n*=155)	Pre-PCV10 (*n*=77)	Post-PCV10 (*n*=79)	
		N (%)	N (%)	N (%)	N (%)	N (%)
Macrolide^*a*^	*mef*	2 (1)	6 (4)	1 (1)	1 (1)	10 (2)
	*erm*B	15 (10)	15 (10)	2 (3)	3 (4)	35 (8)
	*erm*B +*mef*	2 (1)	11 (7)	0 (0)	1 (1)	14 (3)
	*rpl*D2^*b*^	0 (0)	1 (1)	0 (0)	0 (0)	1 (0.2)
Cotrimoxazole^*c*^	*fol*A only	6 (4)	3 (2)	3 (4)	2 (3)	14 (3)
	*fol*P only	24 (15)	19 (12)	12 (16)	10 (13)	65 (14)
	*fol*A +*fol*P	104 (67)	59 (38)	27 (35)	24 (30)	214 (46)
Tetracycline	*tet*O	0 (0)	0 (0)	0 (0)	2 (3)	2 (0.4)
	*tet*M	24 (15)	47 (30)	11 (14)	15 (19)	97 (21)
	*tet*S +*tet*M	0 (0)	1 (1)	0 (0)	0 (0)	1 (0.2)
Chloramphenicol^*d*^	*cat*	1 (1)	0 (0)	1 (1)	1 (1)	3 (1)
Rifampin^*e*^	*rpo*B1	0 (0)	1 (1)	2 (3)	0 (0)	3 (1)
Fluoroquinolone^*f*^	*par*C	1 (1)	0 (0)	1 (1)	1 (1)	3 (1)

*a*, *mef*, macrolide efflux pumps gene resistance; *erm*B, macrolide erythromycin methylation.

*b,* One rare substitution (E7K) within *rpl*D2 gene core genome mutations conferred erythromycin resistance.

*c*, One to five substitutions within the *fol*A gene (Q1H, D2N, V3I, D12T, E14D or I20L) or one to two codon insertions within the *fol*P gene (at nucleotides 169, 174, 176, 177, 178, 180, 182, 185, 186, 188, 189 or 195) result in an intermediate phenotype (MIC 1–2 mg l^−1^) against cotrimoxazole. The *fol*A substitutions (Q1H, Q1Y, D2N, V3I, V6A, Q11H, D12G, E14D or I20L) combined with *fol*P insertions (at the nucleotides 169, 175, 176, 177, 178, 179, 180, 182, 187, 186, 189 or 195) result in a resistant phenotype (MIC ≥4 mg l^−1^).

*d*, *cat*, Chloramphenicol acetyltransferase.

*e*, One substitution within the *rpo*B1 gene (P15A, H21N or K22N) in each isolated.

*f,* One substitution within the *par*C gene (S79C, S29F, or S79Y) in each isolated.

This study observed MDR in 57 (12.2 %) pneumococcal isolates, 22 (9.5 %) in the pre-PCV10, and 35 (14.9 %) in the post-PCV10 period (Table 3). Figs 1 and 2 illustrated the overall resistance among the GPSCs in each age group. The <5 years old group presented higher levels of MDR associated mainly with GPSCs 1, 10, 23, 47 and 341. Focusing on the post-PCV10 period, 12 isolates were associated with the lineage GPSC1 (CC320, serotype 19A) with a profile of high resistance to penicillin (MIC=4 mg l^−1^) plus resistance to cotrimoxazole, erythromycin, and tetracycline; and five related to GPSC47 (ST386, serotype 6C) with a profile of lower resistance to penicillin (MIC=0.125 mg l^−1^) and resistance to erythromycin and tetracycline.

### Pilus islets

**Fig. 4.**
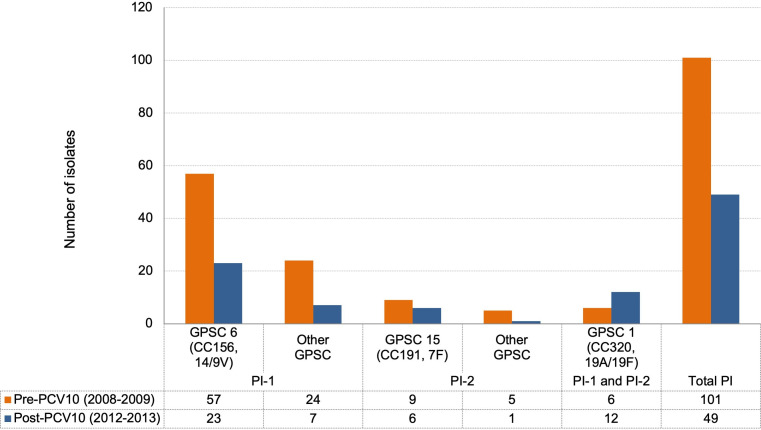
Distribution of pilus islets genes in the main GPSCs by vaccine period studied (*n*=150).

During the study period, the presence of pilus islets (PIs) was observed in 32 % (*n*=150/466) of isolates (pre-PCV10: *n*=81 PI-1, *n*=14 PI-2, and *n*=6 PI-1 and PI-2; and post-PCV10: *n*=30 PI-1, *n*=7 PI-2, and *n*=12 PI-1 and PI-2). The PI-1 type was related to GPSC6 (CC156, serotypes 14/9V) lineage. The combination of P-1 and P-2 was observed only in the lineage GPSC1 (CC320, serotypes 19A/19F) (Fig. 4). In 59 % (*n*=89/150) of isolates with a pilus we observed high level penicillin resistance, 75 isolates presented PI-1 [MIC=2 mg l^−1^; GPSC6 (CC156, serotype 14)] and 14 isolates presenting PI-1and PI-2 (MIC=4 mg l^−1^; GPSC1 (CC320, serotype 19A), with 13 of them also MDR (cotrimoxazole, erythromycin and tetracycline).

## Discussion

Our study analysed the genomic characteristics of a select subset of invasive pneumococcus strains obtained through national laboratory-based surveillance in Brazil. The distribution of serotypes predicted by our WGS study represented as closely as possible the national serotype distribution in the pre- (2008–2009) and post-PCV10 (2012–2013) periods. As expected, a lower prevalence of VT in the post-PCV10 period was due to the substantial PCV10 impact on IPD in the country [28, 29] suggesting that even in a short period after the introduction of the vaccine, it was possible to observe the phenomenon of herd immunity since this VT reduction was also observed in the group of ≥5 years old age, who are not targeted for PCV10 vaccination. However, some increase in NVTs was also documented. A previous Brazilian study [9] analysed a large collection (*n*=8971) of IPD isolates and compared the prevalence of VT and NVT over a longer period, pre-PCV10 (2005–2009) and post-PCV10 (2010–2015), and showed a large IPD VT reduction among children and adult population, documenting a direct and indirect vaccine effect. They also showed a change to NVT as the main cause of the IPD in the post-PCV10 period and concluded that in Brazil there is evidence of cross-protection between serotypes 6B/6A, a fact not observed among the serotypes 6B/6C and 19F/19A. In the post-PCV10 period, our study observed the NVT 3, 19A, 6A, 12F and 6C in the <5 years old age group, and serotypes 12F, 3, 8 and 9 N in the ≥5 years old age group; suggesting, as observed by Brandileone *et al.* [9], an absence of cross-protection between the serotypes 6B and 19F present in the PCV10 composition and the serotypes 6C and 19A, respectively, and revealing that the burden of pneumococcal disease could be further reduced in the country with the introduction in the national childhood immunization programme of PCV13 or other new generation of PCVs (new-PCV10, PCV15 and PCV20), which include the serotypes 3, 6A (with cross-protection to serotype 6C), and 19A in their composition.

Bacterial molecular typing is essential in the surveillance of infectious diseases [20]. This study characterized the baseline population pneumococcal structure for continued vaccine impact monitoring using whole-genome sequencing. Genome data not only allow us to extract public health-relevant data (e.g. serotype and antibiotic resistance profile) from a single experiment but also delineates genetic lineages using both whole-genome clustering method (GPSC) and multi-locus sequencing typing (MLST). Our findings showed a good concordance between these two typing methods. The whole-genome clustering method has further revealed the relationships between strains over a longer timescale by accounting for genetic variations across the whole genome [14]. The GPSCs characterization of the 466 IPD isolates presented a similar genetic structure to the globally GPSC described by the GPS project [14] with the majority of the isolates belonging to these previously described GPSCs. The study collection showed the majority of the GPSC lineages belonged to NVT lineages and a smaller proportion of lineages expressing both VT and NVT serotypes. The post-PCV10 period was marked by the increase in NVT lineages for both the <5 and ≥5 years old age groups. We observed the emergence of NVT lineages, GPSC1 (CC320, serotype 19A), GPSC12 (CC180, serotype 3) and GPSC51 (CC458, serotype 3) in <5 years old and GPSC3 (CC53, serotype 8 and CC62, serotype 11A) in ≥5 years old age groups, highlighting the importance of expanding the PCV coverage for a higher valence vaccine such as PCV13 or others that are still in development (new-PCV10, PCV15 and PCV20) would be useful to further reduce pneumococcal diseases in Brazil.

The pneumococcal population presents several strategies that allow the maintenance of a lineage in the face of pressures imposed through the use of vaccines or antimicrobials: (i) the replacement of a VT by NVT lineage through capsular switching to avoid vaccine-induced immunity, (ii) the acquisition of resistance genes and (iii) the expansion of an existing NVT lineage by filling the open niche left by VT lineage [3, 4]. In our study, we showed the lineage GPSC6 (CC156, serotypes 14–9V) associated with penicillin resistance (PBP 15-12-18 and 45-12-63) was frequent in both vaccine periods and age groups studied. This lineage, well documented in Brazil and globally, is often MDR [30–34], and as expected for a VT lineage [9, 35, 36], showed a decreasing trend, primarily in the <5 years old. A few lineages associated with NVTs in this study have also been documented globally in the post-pneumococcal vaccine periods [14, 37], for example, GPSC1 (CC320, serotype 19A), GPSC3 (CC53, serotype 8), GPSC12 (CC180, serotype 3) and GPSC16 (CC66, serotype 9 N). CC320 serotype 19A was one of the predominant emerging lineages in PCV7 and PCV10 countries [38–43]. Its expansion has been associated with capsular switch events from serotype 19F to 19A and the association with MDR has further allowed its selection in the post-PCV period [41, 43–48]. This lineage was been detected in Brazil since the pre-PCV10 period, but it increased after the PCV10 introduction independent of the age group [49–51]. Another important lineage disseminated worldwide is GPSC12 (CC180, serotype 3), recently associated with MDR [52–54]. In Brazil, this lineage has expanded as a predominant cause of serotype 3 invasive disease in the post-PCV10 era among adults [9, 36]. In this study, GPSC12 was associated with antimicrobial susceptibility, but at IAL has rarely been identified in some isolates as MDR in chloramphenicol, erythromycin, clindamycin and tetracycline (IAL unpublished data). The serotype 8 GPSC3 (CC53) lineage is widely distributed [37, 53, 55, 56] and has been observed in Brazil before PCV introduction [36, 57, 58]. In many countries, the GPSC16 (CC66) is primarily a serotype 9 N lineage associated with carriage [56] but has also been reported with different serotypes [56]. In Brazil and our study, this lineage is mostly invasive isolates expressing the capsular VTs 14 and 19F [31, 33, 36] and rarely are found as serotype 9 N.

The molecular characterization of isolates enabled the identification of several possible capsular switching events from VT to NVT. Initial molecular studies in the serotype 6C reported its origin related to independent recombination events involved isolates from serotype 6A [59], but after that other studies reported possible recombinant events from other serotypes like 6B [60, 61] suggesting multiple genetic origins for serotype 6C. In our study, we estimated a switch from serotype 6B to 6C occurred in the GPSC47 (ST386) before vaccine implementation with an expansion of the serotype 6C clone in the post-PCV10. This clonal expansion correlates with previous data from Lo *et al.* [37] that suggests serotype replacement is mostly mediated by expansion of NVT within VT lineages following vaccine implementation.

The use of PCVs in routine immunization has resulted in a significant effect on the prevalence of antimicrobial resistance, as their formulations include serotypes mostly associated with penicillin and multidrug resistance [62–64]. Beta-lactams are widely used and generally effective for the treatment of pneumococcal infections. Penicillin is recommended to treat non-meningitis pneumococcal infection caused by strains with penicillin MIC <8 mg l^−1^, instead of broad-spectrum antimicrobials such as the third-generation cephalosporin [65]. In Brazil, the use of third-generation cephalosporins is the standard choice for the empiric treatment of meningitis independent of the antimicrobial susceptibility testing results and the combination of cephalosporin and vancomycin has been used in cases of failure to respond to initial treatment. In the present study, we observed significant reductions in penicillin and cotrimoxazole resistance rates and increases in the frequency of tetracycline resistance in the post-PCV10 period for the <5 years old group. We identified resistance determinants by WGS commonly conferring resistance to penicillin, macrolides, cotrimoxazole, tetracycline and chloramphenicol [45]. In concordance, a recent Brazilian study [2] observed a reduction of isolates expressing penicillin MIC ≥0.125 mg l^−1^ in the first 3 years of post-PCV10 introduction (2011 to 2013), with high rates of cotrimoxazole non-susceptibility found during the study years (2007 to 2019), but showing a declining trend after PCV10 implementation, and a gradual increase of non-susceptibility to erythromycin and tetracycline over the study, reaching high rates in the years 2017–2019. We demonstrated that the most frequent lineages related to MDR in the post-PCV10 were NVT GPSC1 (CC320, serotype 19A) with high resistance to penicillin (MIC=4 mg l^−1^), cotrimoxazole, erythromycin and tetracycline, and the single lineage with the presence of the pilus islet PI-1 and PI-2; and the GPSC47 (ST386, serotype 6C) with lower resistance to penicillin (MIC=0.125 mg l^−1^) and resistance to erythromycin and tetracycline. We recommend continued genomic surveillance for long-term monitoring following data presented by Brandileone *et al.* [2] showing how the early impact of PCV10 in reducing non-susceptibility to beta-lactam antibiotics was eroded by increases in penicillin resistance, mainly associated with NVT *S. pneumoniae *, and reaching the highest rates in the years 2017–2019.

In addition to the capsular polysaccharide associated with nasopharyngeal colonization, studies show that *S. pneumoniae* has pilus structure that is involved in the adhesion and invasion of the bacteria in human respiratory epithelial cells [66, 67]. Some studies demonstrated the association of antimicrobial resistance and pili presence, suggesting the pili structure may have a role in the spread of these antimicrobial-resistant lineages [66, 68, 69]. One-third of our isolates had target sequences for the pilus with the majority PI-1 type (74%) and associated with penicillin resistance GPSC6 (CC156, serotype 14) lineage. A recent review [66] analysing the role of the pilus islet in *S. pneumoniae* showed similar overall rates of pili with the predominance of PI-1, presence of PI-1and PI-2 in CC320 serotype 19A lineage, and the association of these genes with antimicrobial and MDR. The presence of the pilus islets PI-1and PI-2 in the MDR GPSC1 (CC320, serotype 19A) lineage may provide an additional advantage for these isolates as they are thought to enhance adherence and colonization [66]. The fact that this lineage is also primarily MDR could explain the success in the establishment of this NVT lineage in the post-PCV10 period.

Despite the limitation that our WGS study was only performed on a subset of the invasive isolates from the pneumococcal laboratory-based surveillance system in Brazil, we did show that the sampling was representative of the overall collection of isolates. We also provided serotype data available on the entire collection and used the subset to identify major antibiotic resistance mechanisms and important genetic lineages in the post-PCV10 period, highlighting the importance of specific NVT genetic lineages in the post-PCV10 period.

Even with the global widespread use of the PCVs, *S. pneumoniae* remains a major bacterial cause of community-acquired pneumonia [70] and one of the main bacterial agents associated with viral co-infections. Since the first great influenza pandemic in 1918 [71], followed almost a century later in 2009 by H1N1 [72] and currently the worldwide COVID-19 pandemic [73] highlights continued surveillance and monitoring of *S. pneumoniae* as a priority. This study provides detailed genomic data of invasive pneumococcal isolates from national surveillance in Brazil, generating a baseline that can help for the creation of long-term surveillance to monitor the vaccine impact and public health strategies.

## Supplementary Data

Supplementary material 1

Supplementary material 2
