# Supplementary material for: Genomic surveillance of invasive Streptococcus pneumoniae isolates in the period pre-PCV10 and post-PCV10 introduction in Brazil
Source: Microb Genom. 2021 Oct 5;7(10):000635. doi: 10.1099/mgen.0.000635 (PMC8627213; doi:10.1099/mgen.0.000635)
Supplement: Supplementary material 1 [file mgen-7-0635-s001.pdf]

Table S1- Distribution of 466 invasive *S. pneumoniae* isolates with whole-genome sequence data included in the study by the Brazilian States

States	Pre-PCV10 (2008-2009) N=232	Post-PCV10 (2012-2013) N=234	Total N=466
São Paulo	114	135	249
Bahia	22	13	35
Minas Gerais	18	12	30
Paraná	15	14	29
Goiás	18	6	24
Rio Grande do Sul	2	19	21
Pernambuco	7	5	12
Ceará	4	7	11
Amazonas	4	5	9
Distrito Federal	4	4	8
Espirito Santo	2	6	8
Mato Grosso do Sul	4	2	6
Santa Catarina	4	2	6
Pará	5	0	5
Rio de Janeiro	3	1	4
Rio Grande do Norte	3	0	3
Mato Grosso	1	1	2
Paraíba	1	1	2
Acre	1	0	1
Alagoas	0	1	1

Table S2. *S. pneumoniae* invasive isolates (N=466) by age groups, clinical diagnosis, and vaccine periods, Brazil

Age group	Epidemiologic data	Vaccine period		Total N=466
		Pre-PCV10 (2008-2009) N=232	Post-PCV10 (2012-2013) N=234	
Age <5 years old	Total	155 (100%)	155 (100%)	310
	Mean age	1.2 years old (1 month-4 year)	1.5 years old (1 month-4 year)	-
	Clinical diagnosis: Meningitis	85 (55%)	66 (43%)	151
	Bacteremia	27 (17%)	51 (33%)	78
	Pneumonia	43 (28%)	37 (24%)	80
	Abscess	0	1 (1%)	1
Age ≥5 years old	Total	77 (100%)	79 (100%)	156
	Mean age	36 years old (5-97 years)	44 years old (5-96 years)	-
	Clinical diagnosis: Meningitis	52 (68%)	29 (37%)	81
	Bacteremia	16 (21%)	37 (47%)	53
	Pneumonia	9 (12%)	13 (16%)	22

Table S3 – Distribution of predicted serotypes, sequence type (ST), and GPSC by age group and vaccine period for 466 sampled invasive isolates from Brazil

Serotype (n)	ST	GPSC[a]	Age <5 years		Age ≥5 years		Total (N=466)
			Pre-PCV10 (n=155)	Post-PCV10 (n=155)	Pre-PCV10 (n=77)	Post-PCV10 (n=79)	
1 (6)	304	31	3	0	2	0	5
	615	2	0	0	0	1	1
3 (39)	180	12	4	7	9	6	26
	232	83	0	0	3	0	3
	260	83	0	0	1	0	1
	458	51	1	5	0	1	7
	11332	51	0	2	0	0	2
4 (9)	770	249	0	1	3	2	6
	7026	70	0	0	2	0	2
	12500	249	1	0	0	0	1
5 (10)	289	8	3	2	0	5	10
6A (19)	471	13	0	0	1	0	1
	473	13	0	2	0	0	2
	724	231	0	1	0	0	1
	1876	13	0	1	0	1	2
	3787	391	2	0	0	0	2
	4269	213	0	1	0	0	1
	4598	24	1	1	1	0	3
	5833	382	1	0	0	0	1
	5847	231	0	0	1	0	1
	11305	23	1	0	0	0	1
	11310	29	0	1	0	0	1
	11312	393	0	1	0	0	1
	13833	391	0	0	0	1	1
	13870	393	0	1	0	0	1
6B (36)	90	23	5	0	0	0	5
	315	47	3	1	0	0	4
	386	47	2	0	0	0	2
	497	13	1	0	0	0	1
	724	231	3	2	0	1	6
	750	37	1	0	0	0	1
	751	37	2	1	0	0	3
	4977	37	0	1	0	0	1
	4978	37	2	1	0	0	3
	5847	231	1	0	0	0	1
	11315	47	0	0	1	0	1
	11787	37	0	1	0	0	1
	12465	231	0	0	1	0	1
	12495	23	1	0	0	0	1
	12514	37	1	0	0	0	1
	12841	37	1	0	0	0	1
	13866	115	1	0	0	0	1
	13872	231	0	0	0	1	1
	14471	811	1	0	0	0	1
6C (10)	386	47	0	5	0	0	5
	1390	111	0	1	0	0	1
	2777	5	0	2	0	1	3
	3930	178	0	1	0	0	1

Serotype (n)	ST	GPSC[a]	Age <5 years Pre-PCV10 (n=155)	Age <5 years Post-PCV10 (n=155)	Age ≥5 years Pre-PCV10 (n=77)	Age ≥5 years Post-PCV10 (n=79)	Total (N=466)
7C (3)	737	16	1	2	0	0	3
7F (15)	191	15	4	2	5	4	15
8 (10)	53	3	0	1	1	6	8
	404	98	1	0	0	0	1
	12574	3	0	1	0	0	1
9N (11)	66	16	0	4	1	6	11
9V (10)	156	6	0	2	1	2	5
	162	6	0	1	2	0	3
	280	43	0	1	0	0	1
	13929	61	0	1	0	0	1
10A (4)	742	311	0	3	0	0	3
	13930	311	0	1	0	0	1
11A (8)	62	3	0	2	1	3	6
	4883	269	0	0	0	1	1
	13871	11	0	1	0	0	1
12F (16)	218	32	1	6	3	4	14
	8102	575	0	0	0	1	1
	8376	289	0	0	1	0	1
13 (6)	761	392	0	1	0	0	1
	11327	289	1	1	0	2	4
	12476	392	0	0	1	0	1
14 (90)	15	18	1	0	0	0	1
	66	16	5	2	2	0	9
	156	6	41	12	7	5	65
	796	571	1	0	0	0	1
	1556	6	1	0	0	0	1
	2335	6	1	0	0	0	1
	12487	16	2	0	0	0	2
	12505	6	1	0	0	0	1
	12506	6	2	0	0	0	2
	12507	6	1	0	0	0	1
	12508	6	1	0	0	0	1
	12837	16	1	0	0	0	1
	12839	6	1	0	0	0	1
	13931	18	0	1	0	0	1
	13932	6	0	1	0	0	1
	13933	6	0	1	0	0	1
15A (12)	63	9	0	0	0	1	1
	67	16	1	0	0	0	1
	73	16	0	0	1	2	3
	1228	11	0	1	0	0	1
	2216	16	0	1	0	0	1
	4913	16	0	3	0	0	3
	10404	702	0	1	0	0	1
	13878	16	0	0	0	1	1
15B/15C (7)	199	4	0	1	0	0	1
	338	5	0	1	0	0	1
	766	131	1	1	0	0	2
	1262	11	0	1	0	0	1
	13869	131	0	0	0	1	1
	13875	131	0	0	0	1	1

Serotype (n)	ST	GPSC[a]	Age <5 years		Age ≥5 years		Total (N=466)
			Pre-PCV10 (n=155)	Post-PCV10 (n=155)	Pre-PCV10 (n=77)	Post-PCV10 (n=79)	
16F (8)	2258	18	0	0	2	0	2
	7027	18	1	1	0	0	2
	7438	386	0	0	0	1	1
	12836	18	0	0	1	0	1
	13867	18	0	1	0	0	1
	14432	18	0	1	0	0	1
17F (2)	739	49	0	2	0	0	2
18A (6)	241	95	1	2	0	2	5
	5063	95	0	0	1	0	1
18B (3)	193	11	2	0	0	0	2
	2814	11	0	1	0	0	1
18C (14)	193	11	7	0	2	1	10
	280	43	0	1	0	0	1
	1358	11	1	0	0	0	1
	12517	11	0	0	1	0	1
	12838	11	1	0	0	0	1
19A (37)	199	4	0	1	0	0	1
	202	1	1	0	0	0	1
	276	10	0	2	1	0	3
	320	1	2	10	0	1	13
	667	4	1	0	0	0	1
	733	18	1	0	2	0	3
	1118	204	2	2	0	0	4
	1451	1	0	1	0	0	1
	2878	204	2	1	0	0	3
	2880	204	1	1	0	0	2
	8640	341	1	1	0	0	2
	9793	341	1	0	0	0	1
	9838	204	0	1	0	0	1
	9942	204	1	0	0	0	1
19F (13)	66	16	1	0	0	0	1
	89	198	0	1	0	0	1
	177	44	1	0	1	0	2
	236	1	2	0	0	0	2
	2307	10	0	1	0	0	1
	3013	44	0	0	1	0	1
	11326	1	1	0	0	0	1
	11328	577	0	1	0	0	1
	12510	44	1	0	0	0	1
	12512	44	1	0	0	0	1
	12513	44	0	0	1	0	1
20 (1)	1030	124	0	0	1	0	1
20B (5)	1030	124	0	3	0	2	5
22F (8)	214	61	0	0	0	1	1
	6403	61	0	5	1	1	7
23A (4)	42	7	0	0	0	2	2
	13873	5	0	1	0	0	1
	13874	7	0	0	0	1	1
23B (4)	727	7	0	0	1	1	2
	945	7	1	1	0	0	2

Serotype (n)	ST	GPSC[a]	Age <5 years		Age ≥5 years		Total (N=466)
			Pre-PCV10 (n=155)	Post-PCV10 (n=155)	Pre-PCV10 (n=77)	Post-PCV10 (n=79)	
23F (20)	81	16	1	0	1	0	2
	242	14	0	1	0	0	1
	338	5	5	4	3	1	13
	353	113	1	0	0	0	1
	735	573	1	0	0	0	1
	3163	5	0	0	1	0	1
	12835	5	0	0	1	0	1
24 (4)	72	16	0	2	0	0	2
	230	10	0	1	0	0	1
	12840	16	0	0	1	0	1
28A (2)	494	191	0	0	2	0	2
29 (3)	5406	394	1	1	0	1	3
33B (1)	14437	139	0	0	0	1	1
34 (1)	743	45	0	0	0	1	1
35B (2)	4877	574	0	0	0	1	1
	14531	574	0	1	0	0	1
35C (1)	13868	61	0	0	0	1	1
38 (5)	393	38	0	3	0	0	3
	755	38	1	1	0	0	2
40 (1)	12483	16	0	0	1	0	1

[a]Black, GPSCs previously described by GPS project; red, Brazilian GPSCs 204, 231, 249, 289, 311, 341, 392-394, 571, 573-575, 577, 702 and 811 (n=16); and blue, globally-spreading lineages recognized in the previous GPS study GPSCs 1, 6, 7, 12, 16, 18, 23 and 32 (n=8).

Table S4. PBP types, associated serotypes, and genetic structure of the five most frequent PBP allele combination with the prediction of MIC ≥0.125mg L^{-1} by age groups in the post-PCV10 period (N=234)

Rank	**Age <5 years old**				**Age ≥5 years old**			
	PBP type (1A--2B--2X)	n	Serotype[a]	GPSC (CC)	PBP type (1A--2B--2X)	n	Serotype[a]	GPSC
1st	13--11--16	11	19A	1 (CC320)	15--12—18	4	14, 9V	6 (CC156)
2nd	15--12--18	7	14, 9V	6 (CC156)	45--12—63	3	14	6 (CC156)
3rd	2--53--77	6	6C, 6B	47 (ST386, CC315)	0--1—1	1	23F	5 (CC172)
					13--11—16	1	19A	1 (CC320)
					2--1—1	1	6C	5 (CC172)
					24--27—28	1	15A	9 (CC63)
					36--34—44	1	6A	13 (CC473)
					7--1—300	1	16F	386 (ST7438)
4th	45--12--63	5	14	6 (CC156)				
5th	0--1--1	3	23F	5 (CC172)				
	2--168--265	3	19A	204 (CC62)				
	36--34--44	3	6A	13 (CC473)				

[a]Black, VT or PCV10 serotypes; blue, NVT additional PCV13 serotypes; and red, NVT non-PCV serotypes.

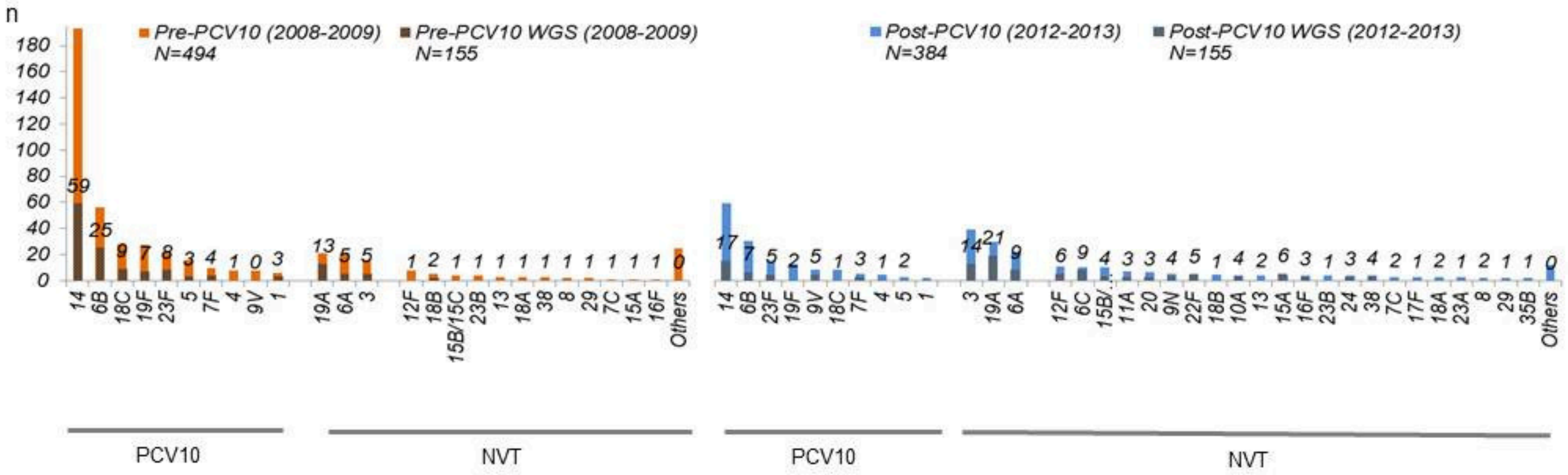

Age group <5 years old

Fig. S1. Distribution of total number of isolates from children <5 years old by serotypes obtained in the laboratory-based surveillance (N=878) compared with the number of isolates characterized by WGS (N=310) in the pre-PCV10 (2008-2009) and post-PCV10 (2012-2013) periods. The numbers indicate the isolates sequenced within each serotype; Others (non-PCV serotypes and non-WGS selected isolates: pre-PCV10 period (n=25) serotypes: 6C (n=4), 35B (n=3), 10A (n=2), 17F (n=2), 20 (n=2), 22F (n=2), 9N (n=1), 11A (n=1), 18F (n=1), 24F (n=1), 35F (n=1) and NT (n=5) and in the post-PCV10 period (n=13) serotypes: 25A (n=3), 11B (n=2), 28A (n=2), 35F (n=2), 10F (n=1), 18F (n=1), 31 (n=1) and 40 (n=1). The NVT is the non-vaccine serotypes including the additional PCV13 serotypes 3, 6A and 19A.

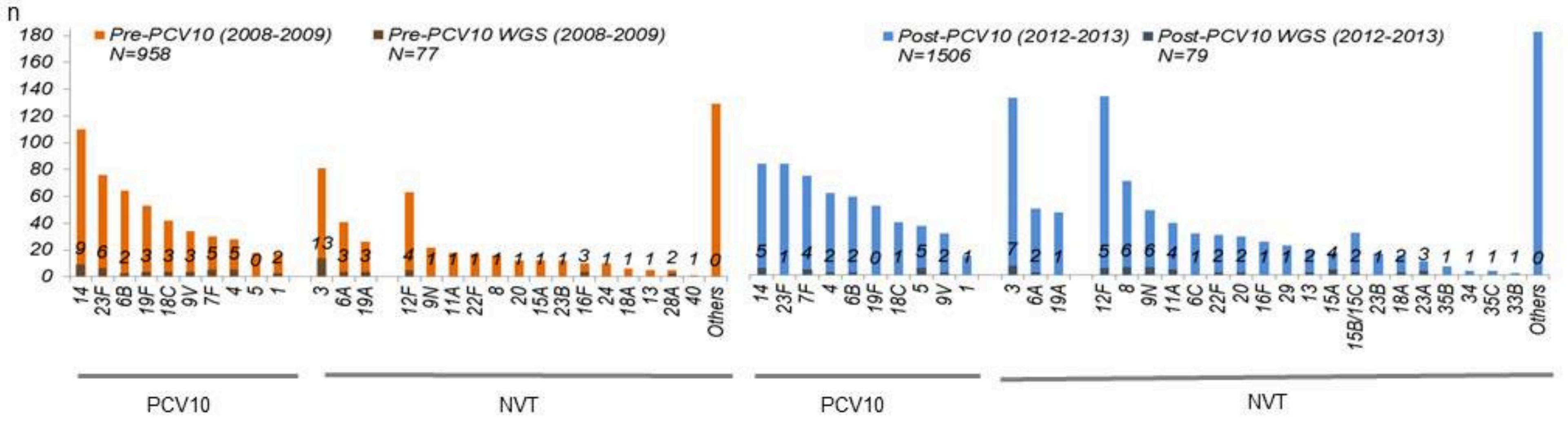

Age group ≥5 years old

Fig. S2. Distribution of total number of isolates from children ≥5 years old by serotypes obtained in the laboratory-based surveillance (N=2,464) compared with the number of isolates characterized by WGS (N=156) in the pre-PCV10 (2008-2009) and post-PCV10 (2012-2013) periods. The numbers indicate the isolates sequenced within each serotype; Others (non-PCV serotypes and non-WGS selected isolates: pre-PCV10 period (n=129) serotypes: 15B/15C (n=21), 6C (n=11), 18B (n=11), 10A (n=9), 7C (n=7), 17F (n=7), 35B (n=6), 35F (n=6), 23A (n=5), 34 (n=5), 29 (n=4), 38 (n=4), 21 (n=2), 7A (n=1), 7B (n=1), 11F (n=1), 28F (n=1), 31 (n=1), 33F (n=1), 35A (n=1), 35C (n=1), NT (n=18) and not serotyped (n=5) and in the post-PCV10 period (n=184) serotypes: 10A (n=35), 17F (n=25), 25A (n=23), 35F (n=20), 7C (n=19), 24F (n=14), 18B (n=13), 31 (n=8), 28A (n=6), 11B (n=3), 21 (n=3), 10F (n=2), 35A (n=2), 40 (n=2), 42 (n=2), 11F (n=1), 19B (n=1) and NT (n=5). The NVT is the non-vaccine serotypes including the additional PCV13 serotypes 3, 6A and 19A.

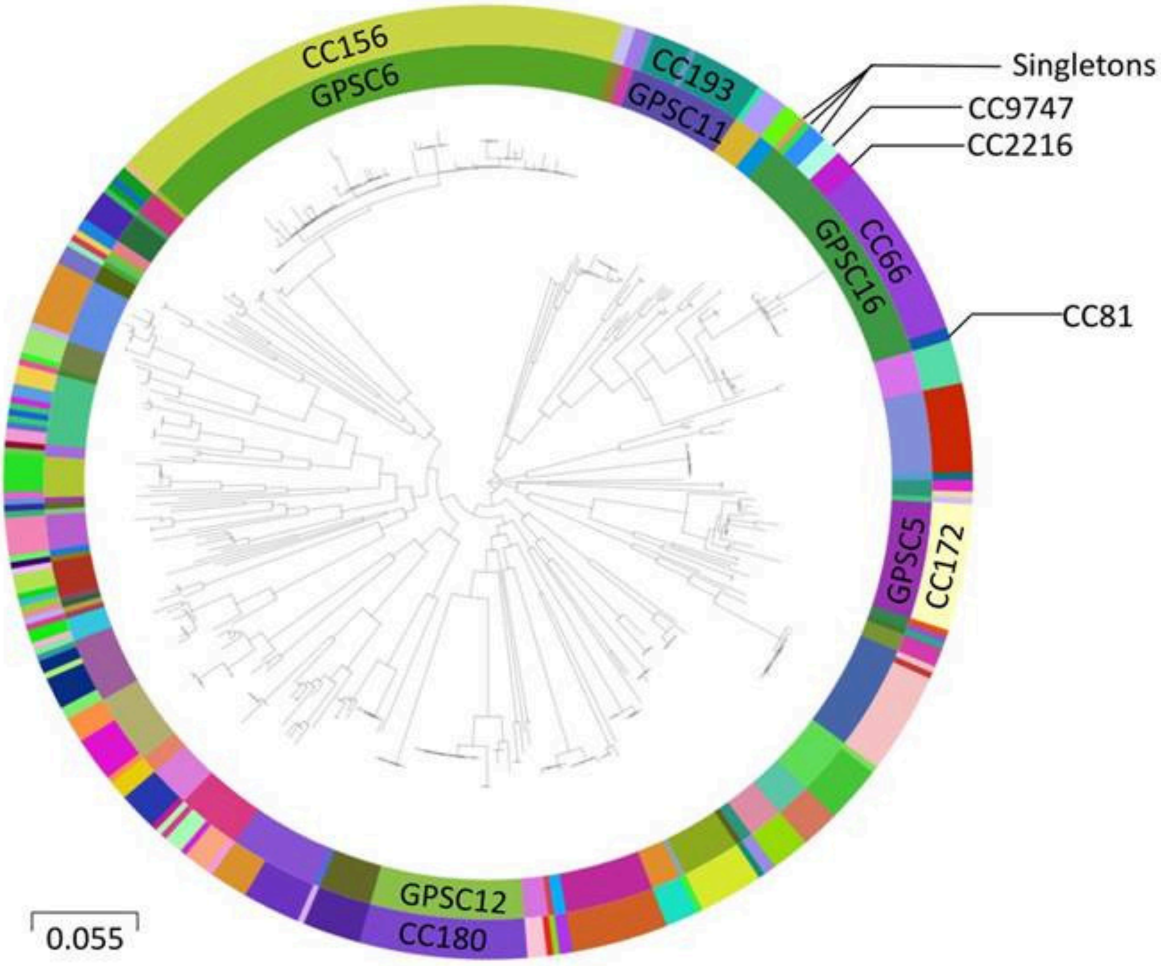

Fig. S3. Phylogeny of 466 studied pneumococcal isolates. The inner ring shows the global pneumococcal sequence clusters (GPSCs) and outer ring clonal complex (CC). The five most predominant GPSCs were labeled. This figure can be visualized at https://microreact.org/project/GPS_Brazil/fd82d3a9.

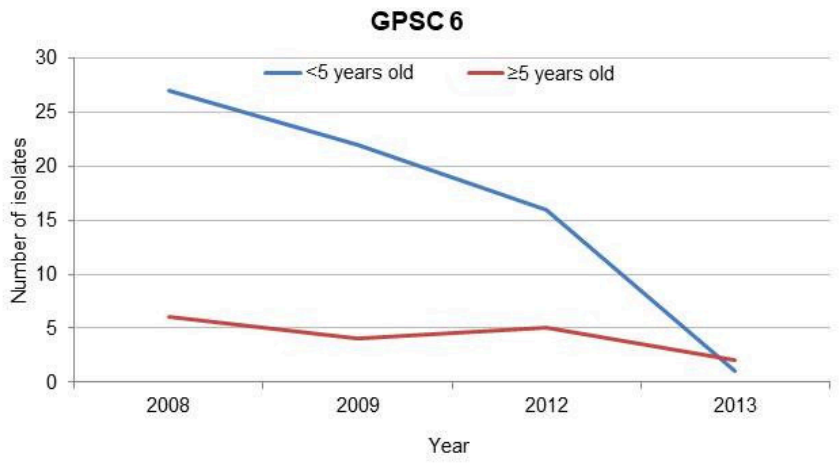

Fig. S4. GPSC6 (CC156) isolates over the study years stratified by age groups.
